# Preliminary evidence from a prospective DTI study suggests a posterior‐to‐anterior pattern of recovery in college athletes with sports‐related concussion

**DOI:** 10.1002/brb3.1165

**Published:** 2018-11-22

**Authors:** Valerie A. Cubon, Murali Murugavel, Katharine W. Holmes, Annegret Dettwiler

**Affiliations:** ^1^ Department of Chemistry and Biochemistry Kent State University at Trumbull Warren Ohio; ^2^ Princeton Neuroscience Institute Princeton University Princeton New Jersey

**Keywords:** diffusion tensor imaging, mean diffusivity, mTBI, radial diffusivity, sports‐related concussion

## Abstract

**Objectives:**

We compared the integrity of white matter (WM) microstructure to the course of recovery in athletes who sustained one sports‐related concussion (SRC), assessing individual longitudinal changes in WM fiber tracts following SRC using pre‐ and post‐injury measurements.

**Materials and Methods:**

Baseline diffusion tensor imaging (DTI) scans and neuropsychological tests were collected on 53 varsity contact‐sport college athletes. Participants (*n* = 13) who subsequently sustained an SRC underwent DTI scans and neuropsychological testing at 2 days, 2 weeks, and 2 months following injury.

**Results:**

Relying on tract‐based spatial statistics (TBSS) analyses, we found that radial diffusivity (RD) and mean diffusivity (MD) were significantly increased at 2 days post‐injury compared to the same‐subject baseline (corrected *p* < 0.02). These alterations were visible in anterior/posterior WM regions spanning both hemispheres, demonstrating a diffuse pattern of injury after concussion. Implicated WM fiber tracts at 2 days include the following: right superior/inferior longitudinal fasciculus; right/left inferior fronto‐occipital fasciculus; right corticospinal tract; right acoustic radiation; right/left anterior thalamic radiations; right/left uncinate fasciculus; and forceps major/minor. At 2 weeks post‐injury, persistently elevated RD and MD were observed solely in prefrontal portions of WM fiber tracts (using same‐subject contrasts). No significant differences were found for FA in any of the post‐injury comparisons to baseline. Plots of individual subject RD and MD in prefrontal WM demonstrated homogenous increases from baseline to just after SRC; thereafter, trajectories became more variable. Most subjects’ diffusivity values remained elevated at 2 months post‐injury relative to their own baseline. Over the 2‐month period after SRC, recovery of WM fiber tracts appeared to follow a posterior‐to‐anterior trend, paralleling the posterior–anterior pattern of WM maturation previously identified in the normal population.

**Conclusion:**

These results suggest greater vulnerability of prefrontal regions to SRC, underline the importance of an individualized approach to concussion management, and show promise for using RD and MD for imaging‐based diagnosis of SRC.

## 1 INTRODUCTION

Accruing scientific evidence regarding both the serious short‐term and potential long‐term consequences of concussive injury in college student athletes has received considerable media attention and raised awareness of concussion as an important public health problem. As such, efforts to identify objective neurobiological correlates of symptom‐based diagnosis of and recovery from concussion have provided a deeper understanding of the physiological and structural consequences of concussive injury.

For example, diffusion tensor imaging (DTI) studies show evidence of structural alterations in college athletes who sustained a sports‐related concussion (SRC) but did not experience a loss of consciousness (LOC) and did not score below 15 on the Glasgow Coma Scale (GCS) (Teasdale & Jennett, 1974). Specifically, in college athletes exhibiting prolonged symptoms (>1 month after SRC) without LOC, increased mean diffusivity (MD) was reported in parts of the left inferior/superior longitudinal and fronto‐occipital fasciculi, the retrolenticular part of the internal capsule, and the posterior thalamic and acoustic radiations (Cubon, Putukian, Boyer, & Dettwiler, 2011). A comparison between measures taken 2 days and 2 weeks post‐injury in varsity contact‐sport college athletes also showed increased radial diffusivity (RD) in a cluster of right hemisphere voxels, spanning the posterior limb of the internal capsule, the retrolenticular part of the internal capsule, the inferior longitudinal fasciculus, the inferior fronto‐occipital fasciculus (sagittal stratum), and the anterior thalamic radiation (Murugavel et al., 2014). These findings are supported by a recent study, conducted as part of the NCAA‐DOD Care Consortium, which found that football players diagnosed with SRC displayed higher MD in frontal and subfrontal white matter (WM) fiber tracts compared to controls within 48 hr post‐injury (Mustafi et al., 2017). Additionally, in the concussed group, a significant positive correlation was found between axial diffusivity (AD) and clinical measures including the Brief Symptom Inventory and the Sports Concussion Assessment Tool (SCAT). Fractional anisotropy (FA) measures were also found to be correlated with Standardized Assessment of Concussion (SAC) performance. The studies presented here highlight increased diffusivity, sometimes accompanied by a corresponding decrease in FA, following SRC. Similar trends are observed in DTI studies on mTBI. For instance, two additional studies (D'souza et al., 2015; Toth et al., 2013) report increased MD and decreased FA during the acute phase of mTBI as compared to age‐ and sex‐matched healthy controls with FA decreases persisting at 1 month (Toth et al., 2013). An additional TBSS study reports increased RD and decreased FA in the subacute phase of mTBI without post‐concussion syndrome as compared to healthy controls with FA decreases persisting at 6 months (Messé et al., 2012). However, there are also literature reports of decreased MD following SRC. For instance, decreased MD, decreased RD, and increased FA were reported during both the acute and chronic phases of SRC (Henry et al., 2011), whereas recent TBSS analyses report widespread decreased MD, RD, and AD during the acute and subacute phases of SRC with decreased MD and RD persisting at 6 months post‐concussion (Lancaster et al., 2016, 2018). Although the directionality of diffusion metrics varies across studies and should be further addressed in future research, previous literature collectively suggests DTI values are abnormal in acute, subacute, and even chronic phases of SRC and mTBI as compared to control populations.

Furthermore, structural differences in the deep WM have been detected in DTI studies assessing the effects of subconcussive blows to the head over the course of an entire season in college athletes playing high‐risk sports (Koerte et al., 2012). For instance, increased RD and AD were found in the right precentral region, corona radiata, and both the anterior and posterior limb of the internal capsule in varsity college ice hockey players when comparing DTI structural metrics derived from pre‐ versus post‐season DTI scans. Additionally, lower cognitive function (CogState30) and decreased fractional anisotropy (FA) in temporo‐occipital WM were found to be associated with a high‐frequency heading rate (>885 – 1,800 headings per year) in adult amateur soccer players (Lipton et al., 2013). Alterations in the WM microstructure have also been observed in contact‐sport college athletes with repeated subconcussive blows to the head, whereas no such changes have been identified in control participants (Bazarian et al., 2014; Lao et al., 2015).

Despite accruing evidence indicating structural and physiological differences after SRC, the course of recovery of WM fiber tracts continues to be poorly understood. This knowledge gap is especially salient when considering the ongoing myelination of the prefrontal portion of WM fiber tracts that continue into the mid‐20s. Although the rate of increase in WM volume in the brain is known to slow after age 10, WM volume has been shown to continue to increase into early adulthood (Giedd, 2004; Iwasaki et al., 1997). A longitudinal MRI study of neurologically normal subjects spanning from children to young adults ranging from 5 to 25 years of age found the rate of WM volume increase to be linear in all four regions of the brain (occipital, temporal, parietal, and frontal) until the mid‐20s (Giedd, 2004; Giedd et al., 1999). While multiple other studies have found myelination to continue into the early to mid‐20s, still others have found myelination to continue for years beyond, until closer to age 30 (Bashat et al., 2005; Benes, Turtle, Khan, & Farol, 1994; Giedd et al., 1999; Giorgio et al., 2008; Lebel, Walker, Leemans, Phillips, & Beaulieu, 2008; Snook, Paulson, Roy, Phillips, & Beaulieu, 2005; Steen, Ogg, Reddick, & Kingsley, 1997). Utilizing histological autopsies, investigators found myelination to continue until age 29, while two separate DTI studies found myelination to occur between the ages of 8 to 27 years and 3 to 30 years, respectively (Benes et al., 1994; Lebel et al., 2008; Snook et al., 2005). Both studies found increases in FA in prefrontal WM tracts, thus indicating WM growth far into the late twenties (Lebel et al., 2008; Snook et al., 2005). Of particular interest is the finding that FA increases along WM fiber tracts in the adult age range were localized to more frontal regions (Benes et al., 1994). Additional studies have reinforced this finding, showing that prefrontal regions tend to develop quite slowly and are the last regions of the brain to become fully myelinated (Barnea‐Goraly et al., 2005; Klingberg, Vaidya, Gabrieli, Moseley, & Hedehus, 1999; Schmithorst, Wilke, Dardzinski, & Holland, 2002; Yakovlev & Lecours, 1967). Taken together, these findings are consistent with the current understanding that the development of WM fiber tracts in the brain follows a posterior‐to‐anterior trend, leaving the prefrontal areas as some of the last brain regions to reach maturation. As a result, prefrontal areas may be more susceptible to concussive injury before full WM fiber tract maturation is reached.

In adolescents, several studies have shown that prefrontal WM fiber tracts are indeed at high risk after mTBI (Babcock, Yuan, Leach, Nash, & Wade, 2015; Borich, Babul, Yuan, Boyd, & Virji‐Babul, 2015; Ewing‐Cobbs et al., 2016; Wozniak et al., 2007). Interestingly, a study involving high school varsity‐level football players found that even without the diagnosis of SRC, post‐season DTI measures demonstrated impaired WM tract diffusivity when compared to pre‐season DTI measures (Davenport et al., 2014). Additionally, a statistically significant linear association was found between the measured total impact and change in WM tract diffusivity (Davenport et al., 2014). Based on evidence previously discussed that prefrontal WM fiber tracts and prefrontal portions of long WM fiber tracts are the last to mature in the normal population, it thus appears important to take the stage of brain development into consideration when evaluating concussive injury.

This study was designed to examine the temporal and spatial course of structural recovery after concussion in college‐aged athletes. In order to elucidate individual differences, this study assessed each athlete's individual pattern of recovery in brain WM during the 2 months after injury using advanced DTI and a baseline, pre‐injury scan. Since the unique use of a baseline scan allows for the examination of specific patterns of individual recovery and structural repair, the results of this study and others based on its findings might, in the future, provide an imaging correlate of neural recovery processes in the deep WM after SRC. Ultimately, this preliminary study might add critical information for an individualized approach to concussion management and prevention of re‐injury.

## 2 METHODS

### 2.1 Participants

All participants were varsity‐level college athletes at Princeton University (Princeton, NJ) in the following sports: football (FB), women's ice hockey (WIH), and men's ice hockey (MIH). A total of 53 right‐handed subjects, 44 males (30 FB and 14 MIH) and nine females (WIH), underwent a pre‐injury baseline MRI scan. All subjects also participated in baseline neuropsychological (NP) testing including the SCAT2, Immediate Post‐Concussion Assessment and Cognitive Testing (ImPACT), Paper–Pencil NP Tests, the Patient Health Questionnaire (PHQ‐9), and the Generalized Anxiety Disorder Test (GAD‐7) (Kroenke, Spitzer, & Williams, 2001; Lovell, Collinsum, Podel, Powell, & Maroon, 2007; McCrory et al., 2009; Spitzer, Kroenke, Williams, & Löwe, 2006). The PHQ‐9 and the GAD‐7 are assessments for depression and generalized anxiety, respectively.

A total of 14 of the 53 subjects enrolled in this study subsequently sustained a sports‐related concussion. These athletes were evaluated by athletic trainers and team physicians within 48 hr post‐injury at University Health Services. The diagnosis of concussion was established using the criteria of the 4th International Consensus Conference on Concussion in Sport (McCrory et al., 2013). Baseline and post‐injury NP testing protocols were identical to those described in our previous study (Dettwiler et al., 2014). All concussed athletes participated in NP testing within 24–48 hr after injury. None of the athletes experienced a loss of consciousness (LOC), and further assessment by the GCS or clinical radiological examination was not warranted. Abnormal performance on NP tests was determined through comparison of post‐injury NP test scores to the participant's baseline scores. Abnormality of ImPACT clinical composites was based on reliable change indices at the 0.8 confidence interval (Iverson, Lovell, & Collins, 2003). Scores on the traditional NP test performance were assessed using Princeton‐specific normative data.

Return‐to‐activity decisions were made by the team physicians. After the athlete was symptom free and their clinical examination, including balance and NP test results, returned to baseline levels, he/she participated in a personalized return‐to‐play progression. Once an athlete was symptom free at rest, had successfully completed the physical activity program, and NP test results were back to baseline, he/she was cleared to return‐to‐play. Follow‐up MRI scans and NP tests at 2 days, 2 weeks, and 2 months post‐injury were performed on all 14 subjects (mean age 20.6 years, standard deviation [*SD*] 1.5, for subjects in Table 1) who sustained a concussion with the exception of subjects missing a time point due to subject unavailability or excessive head motion. One subject was completely excluded from analysis due to excessive motion in the baseline scan. For further details, see Table 1. Participants had no self‐reported history of medical, genetic, or psychiatric disorder and presented without contraindications to MRI. History of concussion was obtained through self‐report. It should be noted that it is difficult to evaluate number of previous concussions objectively in contact‐sport athletes given the limitations and subjectivity of self‐report. The study was approved by Princeton University's Institutional Review Board, and written consent was obtained from all athletes prior to their participation in the study.

**Table 1:** Subject demographics and scanning information

Subject	Age (Years)	Sport	Gender	# Prior concussions	NP normal post‐injury (days)	Symptom free (days)	Return‐to‐play (days)	Days between baseline and injury	MRI inclusion			
									Baseline	2‐day scan	2‐week scan	2‐month scan
1	18	Football	M	0	11	5	16	67	Y	Y	Y	Y
2	23	Ice Hockey	M	5	18	18	DNR^a^	113	Y	Y	Y	Y
3	22	Ice Hockey	F	1	14	11	20	84	Y	N	N	Y
4	20	Football	M	0	18	8	25	386	Y	N	Y	N
5	22	Football	M	0	N/A	21	DNR^a^	63	Y	Y	Y	Y
6	21	Football	M	0	8	4	13	67	Y	Y	Y	Y
7	21	Football	M	0	6	4	8	103	Y	Y	Y	N
8	20	Ice Hockey	M	1	11	7	48	355	Y	Y	Y	N
9	20	Ice Hockey	M	0	15	13	DNR^a^	66	Y	Y	Y	N
10	20	Ice Hockey	F	1	21	12	26	452	Y	Y	N	Y
11	21	Ice Hockey	F	0	14	15	DNR^b^	473	Y	N	Y	Y
12	18	Ice Hockey	M	0	15	5	DNR^b^	161	Y	N	Y	Y
13	22	Ice Hockey	M	0	6	4	11	439	Y	Y	Y	Y

Y: scan included in analysis; N: scan excluded either due to excessive head motion or unavailability of the subject; DNR^a^: subject developed symptoms during return‐to‐play progression and, hence, did not return‐to‐play; DNR^b^: subject did not return‐to‐play, athletic season over; N/A: NP testing did not return to baseline.

### 2.2 Image acquisition

MR images were acquired with a 20‐channel head–neck coil array (Siemens Erlangen, Germany) on a whole‐body 3.0 T Siemens Skyra Scanner. High‐resolution T1‐weighted structural MPRAGE images were acquired at each scanning session (TR/TE/TI = 1.9 s/2.13 ms/0.9 s, matrix size 192 × 256 × 256, voxel size 0.90 × 0.94 × 0.94 mm^3^) with a total anatomical scan time of 4 min 26 s. Diffusion‐weighted images were acquired using a single‐shot spin‐echo pulse sequence with parameters adapted from our previous publication (TR/TE = 9.8 s/96 ms, matrix size 136 × 136 × 70, voxel size 1.88 × 1.88 × 1.9 mm^3^; Cubon et al., 2011). Diffusion data were obtained using a total of 256 gradient directions, each with a b‐value of 1,000 s/mm^2^. In addition, 33 volumes with no diffusion weighting (b = 0) were acquired, yielding a total DTI scan time of 48 min. Subjects watched a movie of their choice during the whole scanning session.

### 2.3 Image analysis

**Figure 1:**
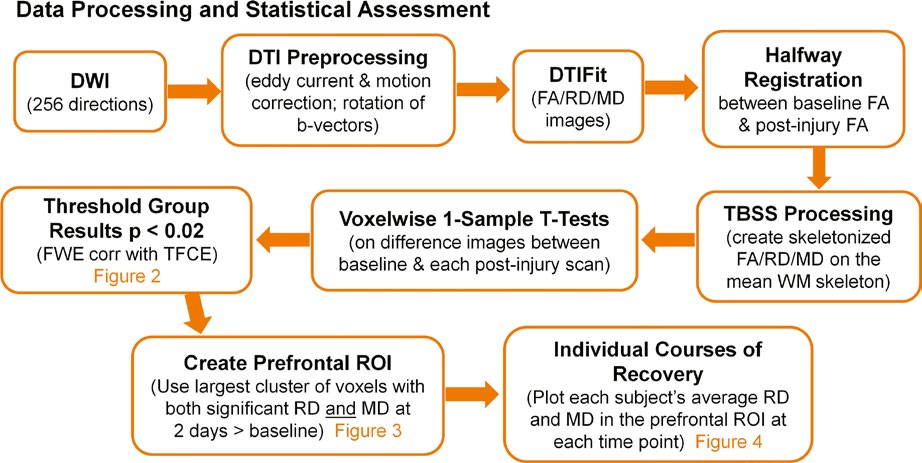
Flow chart of data analysis

For each scan session, the diffusion dataset was concatenated with the 33 B0 volumes and then eddy current and motion corrected using the first B0 volume for reference (FSL software, https://www.fmrib.ox.ac.uk/fsl/, RRID:SCR 002823) (Smith et al., 2004). Images were visually inspected for signal drop‐offs and other imaging artifacts (Figure 1). Scans with excessive head motion were excluded, resulting in nine subjects with 2 days and 2 months post‐injury scans and 11 subjects for the 2 weeks post‐injury scan. B‐vectors were rotated according to the subject's motion estimates before determining the diffusion tensor at each voxel and computing FA, MD, and RD using the FSL subroutine dtifit (Smith et al., 2004). For enhanced individual subject image alignment across time points, a halfway registration was performed between the baseline FA image and each post‐injury FA image for each subject (Madhyastha et al., 2014). Next, a subject‐specific template, created by averaging the two halfway images, was used in TBSS (tract‐based spatial statistics) preprocessing routines to determine the nonlinear transformation to MNI (Montreal Neurological Institute) space (Smith et al., 2006). The resulting transform was then applied to the baseline and post‐injury scans in halfway space, ensuring that both time points receive the same degree of warping. After registration of the halfway images to MNI space, TBSS processing routines were utilized to construct a mean WM tract skeleton representing the centers of all tracts common to all subjects at both time points. The mean WM skeleton was thresholded to include only those voxels with FA >0.25, which excludes regions of high between‐subject variability in the minor tracts. The brainstem and cerebellum were additionally removed from the WM skeleton due to individual subject variability in brain volume resulting in the omission of inferior parts of these structures in a few cases. Each subject's aligned FA images were subsequently projected onto the mean WM skeleton to create a skeletonized FA image (Smith et al., 2006). Skeletonized versions of each subject's MD and RD images were also created using the same registration and projection from the FA data. Skeletonized DTI data (FA/RD/MD) were fed into voxel‐wise one‐sample *t* tests on the difference images between baseline and each post‐injury scan using FSL's randomize program with 5,000 permutations (Smith & Nicholas, 2009). Results were thresholded at *p* < 0.02 (family‐wise error, FWE, corrected with threshold‐free cluster enhancement, TFCE). The two largest clusters from the intersection image of voxels surviving threshold for both RD and MD in the 2 days post‐injury > baseline comparison were used to assess whether anterior and posterior regions display similar percent diffusivity changes immediately following concussion. The largest cluster from the intersection image of voxels surviving threshold for both RD and MD in the 2 days post‐injury > baseline comparison was selected as a region of interest (ROI) to longitudinally evaluate each subject's course of recovery from 2 days to 2 months post‐injury.

## 3 RESULTS

### 3.1 TBSS results of post‐injury to baseline comparisons

#### 3.1.1 2 days post‐injury to baseline

**Figure 2:**
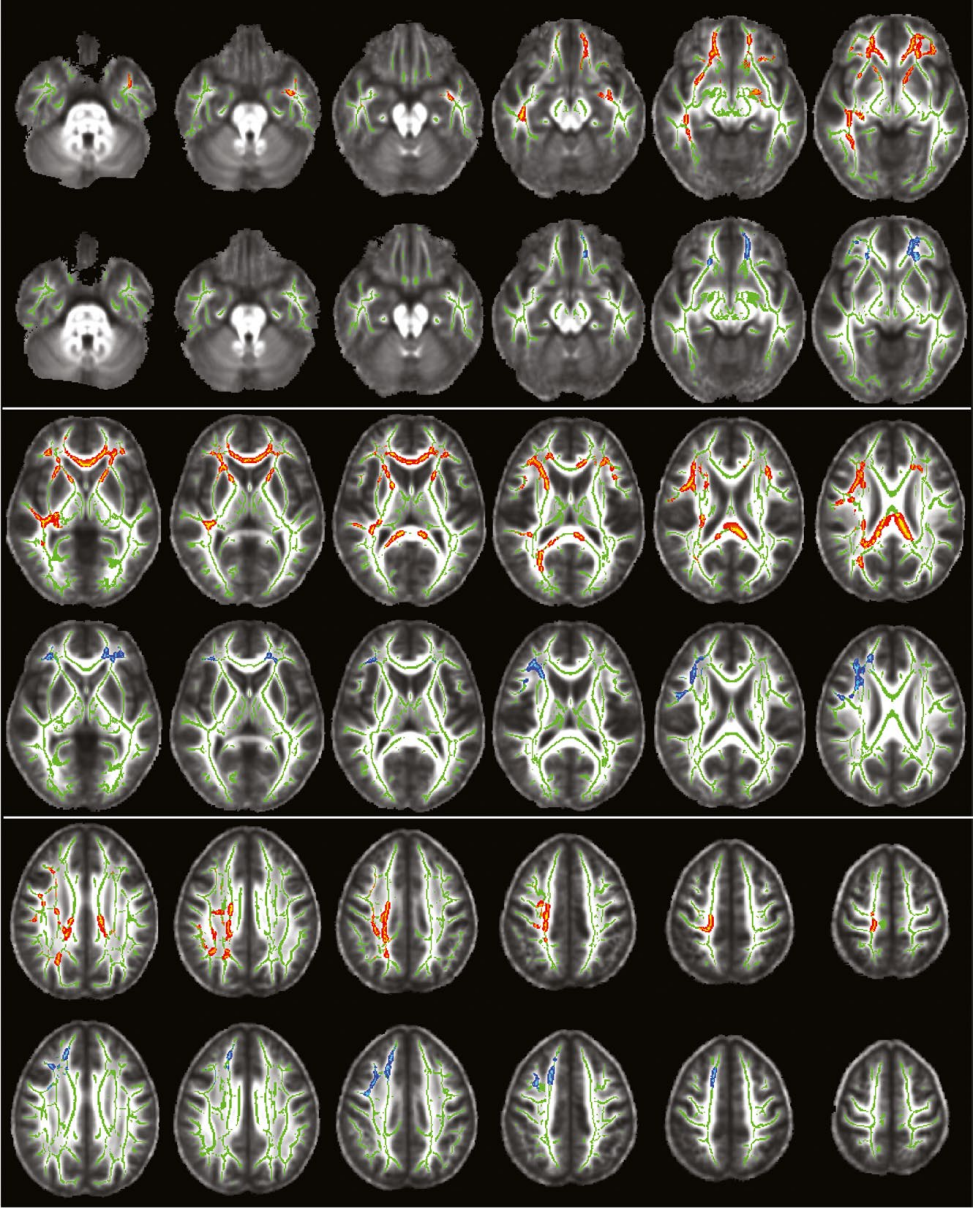
Results of TBSS analyses on the WM skeleton comparing MD and RD at 2 days and 2 weeks post‐injury to baseline. A diffuse pattern of voxels, displayed in red‐yellow, exhibits significantly *higher RD and MD values* (corrected *p* < 0.02) at 2 days post‐injury as compared to baseline. Voxels with significantly *higher RD and MD* (corrected *p* < 0.02) at 2 weeks post‐injury as compared to baseline were observed solely in the prefrontal portion of WM fiber tracts, displayed in blue‐light blue. Voxels are inflated into local tracts and overlaid onto the WM skeleton (green) for ease of visualization. The underlay is the mean FA group volume (grayscale). Images are shown in radiological convention (image right =subject's left) for axial slices every 5 mm from MNI slice *z* = −30 mm (top left image) to *z* = 55 mm (bottom right image)

**Figure 3:**
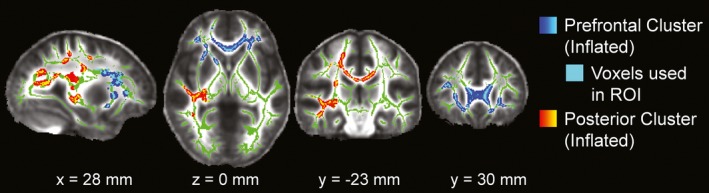
The two largest clusters of voxels with significantly *higher RD and MD values* (corrected *p* < 0.02) from the TBSS analyses comparing 2 days post‐injury to baseline. The largest cluster, located in anterior prefrontal WM, is highlighted in blue‐light blue and is the prefrontal WM ROI subsequently used to longitudinally evaluate each subject's individual course of recovery in Figure 4. Voxels are inflated into local tracts for ease of visualization; however, only the light blue voxels which lie directly on the WM skeleton were used for the ROI. The clusters are overlaid onto the corresponding WM skeleton (green). The underlay is the mean FA group volume (grayscale). Images are shown in radiological convention (image right =subject's left) for MNI slice coordinates: *x* = 28 mm (sagittal view), *z* = 0 mm (axial view), *y* = −23 mm (coronal view), *y* = 30 mm (coronal view)

Tract‐based spatial statistics analyses yielded significantly increased RD and MD (*p* < 0.02, FWE corrected with TFCE) in concussed athletes at 2 days post‐injury as compared to baseline in paired, between‐session *t* tests on the WM skeleton. Voxels with significantly higher values in both RD and MD (12,348 total voxels, each 1 mm^3^) were located in anterior and posterior WM regions spanning across both hemispheres, suggesting a diffuse pattern of injury after one concussive injury (Figure 2, red‐yellow voxels inflated into local tracts for visualization). More specifically, implicated WM fiber tracts include the right superior/inferior longitudinal fasciculus; right/left inferior fronto‐occipital fasciculus; right corticospinal tract; right acoustic radiation; right/left anterior thalamic radiations; right/left uncinate fasciculus; and forceps major and minor. The John Hopkins University (JHU) ICBM‐DTI‐81 WM and JHU WM tractography atlases included in FSL were used to determine the anatomical regions referenced. More regions were affected in the right hemisphere, in particular in the posterior regions of WM fiber tracts. Additional comparisons between anterior and posterior regions (Figure 3 light blue and yellow voxels respectively) revealed similar percent diffusivity changes from baseline with insignificant paired *t* test results. The diffusivity changes from baseline in anterior and posterior regions also correlated across subjects with Pearson *p* = 0.0019 and *p* = 0.0023 for MD and RD, respectively. These results suggest that the initial diffusivity changes at 2 days post‐injury are comparable across anterior and posterior brain regions within each subject.

#### 3.1.2 2 weeks post‐injury to baseline

Persistent significant structural differences (*p* < 0.02, FWE corrected with TFCE) were observed in TBSS analyses comparing RD and MD of concussed athletes at 2 weeks post‐injury to baseline using paired, between‐session *t* tests on the WM skeleton. Voxels demonstrating persistent structural differences with significantly elevated RD and MD (3,375 voxels, each 1 mm^3^) were solely located in the prefrontal portion of WM fiber tracts (Figure 2, blue‐light blue voxels inflated into local tracts), including right/left inferior fronto‐occipital fasciculus, right superior longitudinal fasciculus, right anterior thalamic radiation, right/left uncinate fasciculus, and forceps minor. Interestingly, the shift from widespread structural alterations to more localized portions of WM fiber tracts between 2 days and 2 weeks after injury suggests that the posterior regions of deep WM recover during the first 2 weeks post‐injury, whereas structural differences in the prefrontal segment of WM fiber tracts persist (Figure 2). This suggested posterior‐to‐anterior recovery trend is consistent with the posterior‐to‐anterior maturation of WM fiber tracts in the brain, which is known to continue in the prefrontal cortex into early adulthood (Bashat et al., 2005; Benes et al., 1994; Giedd et al., 1999; Giorgio et al., 2008; Lebel et al., 2008; Snook et al., 2005; Steen et al., 1997). Additionally, superior axial slices reveal an anterior shift of significant voxels to adjacent regions from 2 days to 2 weeks post‐injury (bottom rows in Figure 2) suggesting an ongoing or delayed process is occurring in prefrontal WM during recovery.

#### 3.1.3 2 months post‐injury to baseline

No significant differences (at *p* < 0.02) were found for RD or MD in the 2 months post‐injury to baseline comparisons using paired, between‐session *t* tests on the WM skeleton. However, a trend of increased RD and MD in frontal cortex (at *p* < 0.05) was observed. No significant differences or trends were found for FA in any of the post‐injury comparisons to baseline.

### 3.2 Individual subject courses of recovery for RD and MD from the selected prefrontal ROI

**Figure 4:**
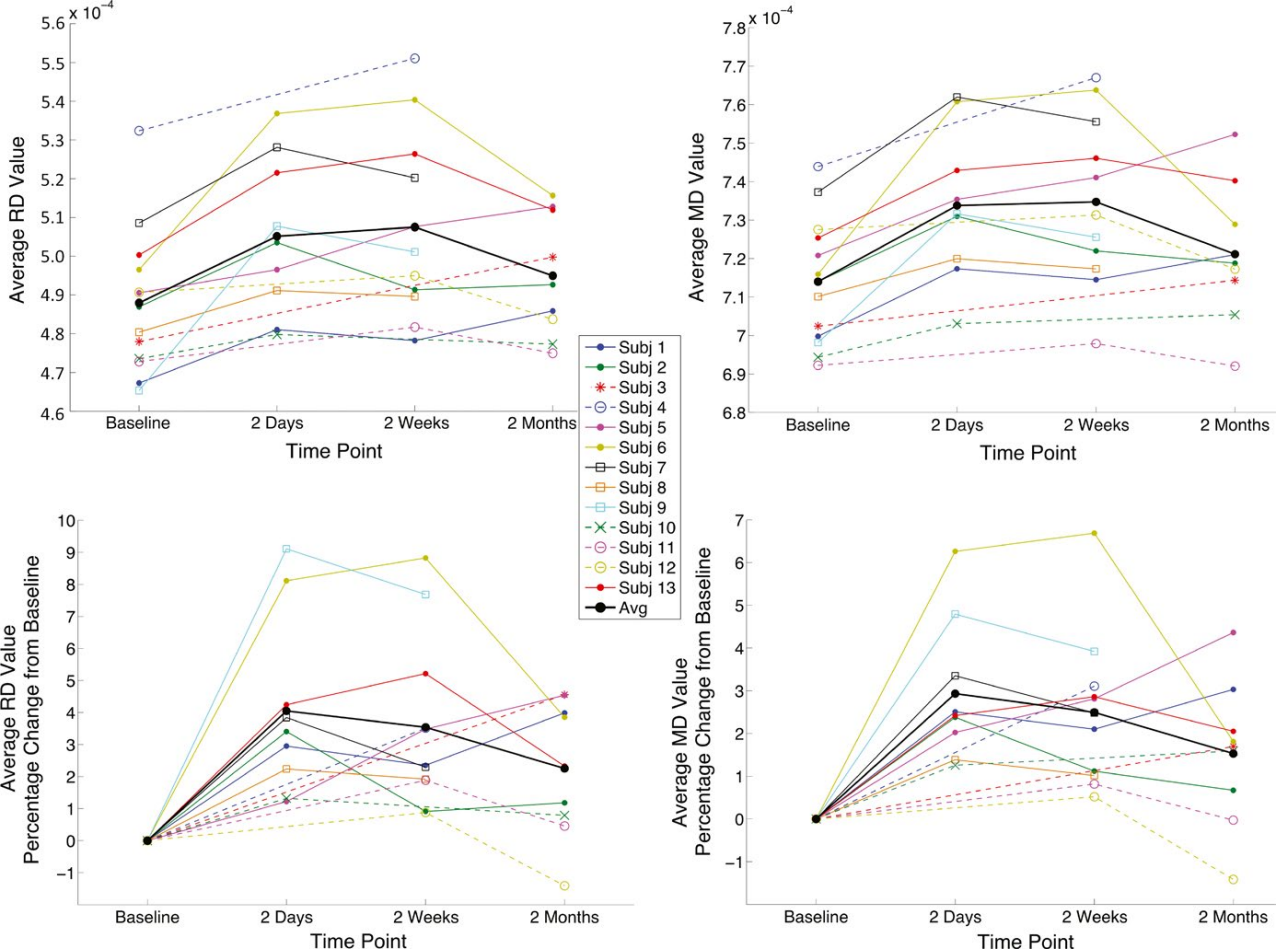
Individual subject RD and MD courses of recovery in prefrontal WM. (Top row): Each subject's average RD and MD value for voxels comprising the prefrontal WM ROI (shown as the light blue voxels in Figure 3) is plotted for each scanning time point. (Bottom row): These same average RD and MD values are plotted as a percentage change from each subject's own pre‐injury baseline scan. From this plot, average RD and MD values in the ROI can be tracked through time from baseline to 2 months for each subject individually. Filled circles indicate athletes who participated in all four sessions (1, 2, 3, and 4); open circles indicate athletes with sessions 1, 3, and 4; boxes indicate sessions 1, 2, and 3; exes indicate sessions 1, 2, and 4; and asterisks indicate sessions 1 and 4. Solid lines connect consecutive measurements, and dashed lines connect nonconsecutive measurements. The black line connects the mean values of the concussed subjects across the four time points

Persistent differences in the prefrontal segment of WM fiber tracts, as revealed in the TBSS analyses, were further evaluated using individual subject data. The largest cluster (5,324 voxels), shown in light blue in Figure 3, of voxels surviving threshold for both RD and MD in the 2 days post‐injury to baseline comparison (*p* < 0.02, FWE corrected with TFCE) was selected as a ROI to longitudinally evaluate each subject's individual course of recovery in prefrontal WM. Plots of individual subject's average DTI values (RD, MD) in the prefrontal ROI at each of the time points collected demonstrate homogeneous patterns of increased diffusivity from baseline in all subjects immediately following concussion (top row Figure 4). These results are further emphasized in plots of percentage change from baseline for each subject's DTI values (bottom row Figure 4). Between the 2 days and 2 weeks post‐injury time points, subject trajectories are more variable and this variability further increases at 2 months post‐injury. This finding strongly suggests that one concussive injury does affect the deep WM fiber tracts. Despite the fact that only a trend (0.025 < *p *< 0.05) was observed in the 2 months to baseline TBSS DTI group comparisons, seven out of nine subjects’ RD and MD values in the prefrontal ROI continue to be above their own baseline values at 2 months post‐injury (paired *t* test *p* < 0.00649 and *p* < 0.0134, for RD and MD, respectively).

## 4 DISCUSSION

Previous longitudinal studies assessing concussion typically evaluate pre‐ and post‐*season* measurements. The current preliminary study, however, investigated pre‐ and post‐*injury* measurements to monitor an athlete's progress toward recovery using DTI. This study revealed diffuse alterations throughout the brain in both posterior and anterior regions of deep WM immediately after only one diagnosed sports‐related concussion and, more importantly, it uncovered a distinctive pattern of brain recovery that indicates a posterior‐to‐anterior progression. Diffusivity differences persisted in the prefrontal segment of WM fiber tracts at 2 weeks and, for some subjects, even 2 months post‐injury, whereas diffusivity values normalized in the posterior segment of WM fiber tracts at both time points. Specifically, the anterior portion of right/left inferior fronto‐occipital fasciculus, right superior longitudinal fasciculus, right anterior thalamic radiation, right/left uncinate fasciculus, and forceps minor demonstrated prolonged increases of both RD and MD, suggesting that these tracts may be more fragile or particularly vulnerable and less resilient to concussive injury. Remarkably, this preliminary finding regarding brain recovery following concussion appears to mirror the sequence of WM fiber tract maturation, suggesting that later maturing brain regions may be more susceptible to concussive injury.

While DTI offers a retrospective view of structural alterations in the WM of the brain, it does not reveal the exact location of impact or the level of g‐force with which the subject was hit. Studies have found that devices that collect those metrics may be useful for identifying concussive blows. For instance, finite element models (FEM) attempt to locate brain region‐specific strain and stress responses to concussion by simulating the biomechanical event using recorded impact kinematics. The collection of accelerometer data for FEM analyses has progressed from laboratory‐based impact reconstructions to actual helmeted and unhelmeted impacts from subject data (McAllister et al., 2012; Patton, McIntosh, & Kleiven, 2013, 2015; Viano et al., 2005; Zhang, Yang, & King, 2004). Additionally, recent research incorporated WM anisotropy from DTI scans into the FEM (Giordano, Zappalà, & Kleiven, 2017). Collectively, these studies agree that peak strains and stresses of concussive injury are located near the brainstem in central brain areas such as the midbrain, thalamus, and corpus callosum regardless of the actual head impact location. However, research has also suggested that head impact sensors do not yet have the sensitivity needed to accurately locate the impact azimuth or determine a reliable g‐force threshold for concussion diagnosis (Allison, Kang, Bolte, Maltese, & Arbogast, 2014; Guskiewicz & Mihalik, 2011; King, Hume, Gissane, Brughelli, & Clark, 2016; Kutcher et al., 2013; O'Connor, Rowson, Duma, & Broglio, 2017). For example, a study seeking to quantify head impact exposure for a collegiate women's soccer team over the course of a season found that head impact sensor data had both a high level of false positives and false negatives when compared to video analysis of potential concussive hits and a medical assessment of concussive injury (Press & Rowson, 2017). These and other similar findings may be accounted for by the fact that sensor data interpretation rests on the assumption that the skull and brain move continuously as one, whereas the brain is actually surrounded by cerebrospinal fluid within the skull and is affixed to the brain stem. Hence, the brain can move within the skull. Given these anatomical facts and the findings described above, the addition of sensor data would, at this point in time, most likely not have broadened our DTI findings.

Examination of subjects’ self‐report of their respective impact locations revealed there was no tendency toward frontal impacts in our subject population. In fact, the site of impact was variable across subjects with most subjects (38%) reporting the side of the head located near temporal regions and not prefrontal areas. Statistical analyses of DTI values in the prefrontal ROI compared to a posterior ROI (displayed as the light blue and yellow voxels in Figure 3, respectively) suggest the observed recovery pattern is characteristic of concussive injury and not merely an artifact of impact location. For instance, paired *t* tests comparing percentage change from baseline values at 2 days post‐injury were insignificant between the prefrontal and posterior ROIs for both MD and RD. Additionally, Pearson correlations between prefrontal and posterior ROI percentage change from baseline values at 2 days post‐injury were significant (*p* = 0.0019 and *p* = 0.0023 for MD and RD, respectively). These two results indicate that the initial pattern of injury immediately following a concussive event exhibits similar diffusivity changes that correlate in both anterior and posterior regions. Taken together, these findings lend further support that the location of impact most likely does not account for the posterior‐to‐anterior pattern of recovery identified in this study.

Two previous DTI studies, assessing either athletes with prolonged symptoms or longitudinal post‐injury differences, also reported structural alterations identified by increased diffusivity in nearly the same posterior anatomical region (sagittal stratum) but in contralateral hemispheres (Cubon et al., 2011; Murugavel et al., 2014). Specifically, RD decreased significantly in a comparison between 2 days and 2 weeks post‐injury in the right sagittal stratum, indicating recovery in this particular brain region (Murugavel et al., 2014). Interestingly, in the current study, similar right hemisphere posterior anatomical regions also demonstrated increased RD and MD at 2 days post‐injury when compared to their own baseline, but not at 2 weeks or 2 months post‐injury. Collectively, these studies suggest that the posterior region recovers within 2 weeks after injury, but may remain abnormal in athletes experiencing persistent symptoms. The current study may have more effectively captured additional regions exhibiting structural alterations due to concussion through the use of a baseline scan. Such areas emerged in the prefrontal segment of WM fiber tracts with differences persisting at 2 weeks post‐injury and even at 2 months post‐injury when evaluating individual courses of recovery. These results further emphasize the importance of a baseline scan and suggest that prefrontal WM diffusivity values may provide the sensitivity needed to monitor the course of recovery with WM fiber tract recovery following a posterior‐to‐anterior trend, reminiscent of the posterior‐to‐anterior pattern that brain development follows. Future studies will be needed to confirm this trend of recovery in college‐aged athletes in addition to studies investigating how WM fiber tract injury and subsequent recovery patterns may be altered at different stages of brain development, namely childhood and adolescence. As such, additional research across other ages and stages of development must also be conducted in order to further understand how concussive injury may interact with WM fiber tract development.

A prior longitudinal study investigated concussive injury using a different imaging modality that assessed pre‐ and post‐injury measurements of myelin water fraction. This study found that concussed athletes demonstrated myelin disruption at both 2 days and 2 weeks post‐injury. However, these values normalized to pre‐season values by 2 months (Wright et al., 2016). Other longitudinal studies typically assess concussion using pre‐ and post‐season measurements with a particular focus on repetitive head impacts. For example, a DTI study comparing pre‐ and post‐season measurements in a group of varsity ice hockey players (mean age 22 years) revealed increased diffusivity values in the right precentral region, right corona radiata, and the anterior and posterior limb of the internal capsule, over the course of one entire season (Koerte et al., 2012). Additionally, heading by amateur soccer players (mean age 30.9 years) was associated with poorer neurocognitive performance on memory tests and abnormal WM microstructure in temporal–occipital regions (Lipton et al., 2013). Taken together, these studies reveal effects of repetitive head impacts rather than monitoring the effects of and recovery from one clinically diagnosed concussion, as was the purpose of the current study. Bearing this in mind, it must be taken into consideration that, in the current study, subconcussive hits experienced during the time from the baseline scan to concussion and once the athlete returned‐to‐play may have contributed to diffusivity changes. However, these contributions would be highly variable and, on average, subjects demonstrated increased diffusivity at 2 days followed by a gradual decline at 2 weeks and 2 months post‐injury (solid black line in Figure 4).

Remarkably, most subjects in the current study were not back to their baseline diffusion values by 2 months in prefrontal WM, an area underlying the lateral and dorsolateral frontal cortical regions. These regions are known to be essential for complex behaviors such as executive control, decision‐making, and impulse control. Recent work looking at subjects from 8 to 26 years of age using diffusion‐weighted imaging (DWI) found a positive correlation between impulse control and age, noting that individuals with higher WM integrity of the frontostriatal tracts demonstrated better delayed gratification (Achterberg, Peper, Duijvenvoorde, Mandl, & Crone, 2016). As WM fiber tracts matured into early adulthood, so too did future delay of gratification skills. A similar DWI study of subjects from 8 to 25 years of age also found that increases in frontal–striatal connections were correlated with improved impulse control, which was also found to be positively correlated with age (van den Bos, Rodriguez, Schweitzer, & McClure, 2015). Given that prefrontal WM tracts mature into the mid‐20s and maturity has been found to be correlated with impulse control, our preliminary findings demonstrating prolonged recovery after injury thus raise concern regarding the consequences concussion may have for the development of executive function.

In light of these relationships, damage to WM fiber tracts in prefrontal regions may have potentially serious long‐term effects. Prominent neurofibrillary tangles (NFT) have been found in isolated perivascular foci within neurons and astrocytes at the base of the sulci limited to the frontal lobe in the early stages (mild pathology stages I and II) of chronic traumatic encephalopathy (CTE; Stein, Alvarez, & McKee, 2015). The stage of CTE has been positively correlated to the number of years an athlete has played American football, suggesting that prolonged duration to repeated blows to the head may lead to CTE (McKee et al., 2013). As of now, there is no method available to determine whether a young athlete will develop CTE and, as such, a link between concussive injuries experienced earlier in life and CTE cannot be inferred. However, given the regions of the brain associated with early stages of CTE, our findings that WM fiber tract recovery was delayed in the frontal regions of the brain are particularly salient. Our preliminary results of the current study as well as earlier studies (Dettwiler et al., 2014; Murugavel et al., 2014) suggest that neural recovery takes much longer than neurocognitive recovery, thus potentially indicating that athletes are returning‐to‐play before full neural recovery is complete. This is most apparent when evaluating individual subject diffusivities over time in prefrontal cortex, highlighting a unique advantage of individualized approaches. The majority of subjects in the current study exhibit diffusivity values that remain above their own baseline value even at 2 months post‐injury (Figure 4) despite the fact that subjects were both symptom free and NP normal. This may put athletes at greater risks of developmental and long‐term effects of concussion. Additional, future longitudinal studies should assess recovery from concussive injury with multiple time points that extend beyond 2 months.

Using a baseline DTI scan greatly enhances detection of the effects of concussion on water diffusivity through the distinct comparison of an individual subject's post‐concussion values to their own pre‐concussion measurements. Consistent increases in water diffusivity across all subjects from baseline to 2 days post‐concussion in prefrontal WM suggest DTI has the potential to capture the hallmarks of concussive injury. Baseline scans, in conjunction with longitudinal post‐concussion scans, heighten the sensitivity of detecting alterations unique to each individual subject. Baseline scans furthermore provide an enhanced alignment of WM fiber tracts and hence control for interindividual differences often confounded in cross‐sectional studies. The use of baseline scans also allows for a deeper understanding of specific patterns of individual recovery and structural repair without the interference of interindividual differences since each subject essentially serves as their own control.

With these data from baseline, 2‐day, 2‐week, and 2‐month scans, the current study was thus able to elucidate prefrontal WM as a brain region that exhibits pronounced increased diffusivity with prolonged variable recovery following concussion in athletes 18–23 years of age. Individual trajectories of WM diffusivity values in this region over time (Figure 4) may assist in evaluating a single subject's progress toward recovery after concussion and identify varying levels of resilience during the early phase of recovery. Thus, an individualized imaging assessment of recovery may, in the future, aid the clinician with the difficult decision for the athlete's return‐to‐play and overall concussion management. Variability in these trajectories across subjects after 2 days post‐concussion may be due to developmental factors, genetic predisposition, differences in the number of previous concussions, or the timing of each athlete's return‐to‐play. This variability further reinforces the importance of an individualized approach to monitor concussion.

A recent longitudinal mTBI study that also utilized an individualized approach reported similar increases in RD and MD that additionally correlated with worse memory performance and worse somatic autonomy (Strauss et al., 2016). Interestingly, the authors suggest that increased RD may serve as a good early predictor of long‐lasting dysfunction (Strauss et al., 2016). Even though post‐injury FA abnormalities were also reported, all of the post‐injury time points in the current study displayed insignificant FA results. These findings are consistent with results of our previous publications where diffusivity values were more sensitive to concussive injury, even across various SRC populations (Cubon et al., 2011; Murugavel et al., 2014).

Substantial evidence by both research previously discussed and the current preliminary study supports the validity and importance of an individualized longitudinal approach with baseline measurements to manage concussion (Strauss et al., 2016). Despite the low subject number in our current study, the DTI data show promise in providing a sensitive method to temporally monitor brain regions which are vulnerable to concussive injury and to assess each athlete's individual path to recovery. Future work should further evaluate water diffusivity in the prefrontal WM ROI as a potential diagnostic marker and as a tool to monitor the progression of recovery following SRC, observing whether it follows the same posterior‐to‐anterior trend noted in this study. Additional studies will need to include comparisons between gender, and other age ranges so as to observe the potentially varying effects of concussion at different stages of brain development. Given that this was a preliminary study, future studies with a larger subject number should also be conducted. One limitation of DTI studies in general is their inability to identify the exact underlying structural cause of any changes in diffusion measures; however, the power of DTI should not be lost by this statement, as DTI values are influenced by structural alterations such as axonal ordering, axonal density, degree of myelination, increased water content due to edema, axonal packing, or results of inflammatory processes (Beaulieu, 2002; Jones, Knösche, & Turner, 2013). Even though TBSS analyses provide enhanced alignment of deep WM fiber tracts across subjects, results are limited to larger bundles of these tracts and potential changes at the gray–white matter junction are not effectively captured. This is a second limitation of DTI in general, and methods to address this issue were not within the scope of the current study and should be investigated in future research. While the current study's subject population did not experience persistent symptoms, future studies should investigate whether subjects with post‐concussive syndrome also show prolonged changes in water diffusivity from baseline in the prefrontal WM ROI. To assess whether structural differences in prefrontal WM fiber tracts are predictive of neurocognitive outcome, correlations of diffusivity values with NP test results and clinical symptoms (symptom score, GAD anxiety score, and PHQ‐9 depression screening) will be investigated and presented in a future publication.

In conclusion, despite its small sample size, the current preliminary study provides a longitudinal pre‐/post‐concussion DTI analysis to monitor recovery processes by tracking diffusion measures from baseline to 2 months after one SRC with no LOC. Within 2 days post‐injury, increased water diffusivity (RD and MD) was widespread throughout the brain in all subjects and was followed by a posterior‐to‐anterior course of recovery reminiscent of brain development patterns. Elevated RD and MD persisted at 2 weeks post‐injury in prefrontal WM, thereby highlighting fiber tracts that may, at this age level, be particularly vulnerable to concussive injury. Moreover, RD and MD remained elevated at 2 months post‐injury in most subjects despite normalization of NP test results, suggesting the recovery process in prefrontal WM was not complete by 2 months. Longitudinal, individualized analyses with baseline pre‐injury scans, such as the current study, show promise in providing an imaging correlate of recovery processes after SRC with the potential of utilizing an individualized approach to concussion management and prevention of re‐injury.

## CONFLICT OF INTEREST

No competing financial interests exist.
